# Annexin–Membrane Interactions Across Eukaryotic Domains of Life—A Comparative Approach

**DOI:** 10.3390/ijms26136517

**Published:** 2025-07-07

**Authors:** Dawid Warmus, Erina Alexandra Balmer, Carmen Faso

**Affiliations:** 1 Institute of Cell Biology, University of Bern, 3012 Bern, Switzerland; 2 Graduate School for Cellular and Biomedical Sciences, University of Bern, 3012 Bern, Switzerland; 3 Multidisciplinary Center for Infectious Diseases, University of Bern, 3012 Bern, Switzerland; 4 Institute for Infectious Diseases, University of Bern, 3001 Bern, Switzerland

**Keywords:** membrane, lipid, annexin, eukaryote, interaction, membrane trafficking, ion channel, protist, plant, animal, parasite, free-living, evolution

## Abstract

This review explores the interaction of annexins with membranes across a variety of eukaryotic domains of life, highlighting this protein family’s role in cellular processes due to its lipid and calcium-binding properties. By comparing annexins’ functions in diverse organisms, we aim to uncover novel insights into their mechanisms of action, particularly in membrane repair, protein trafficking, and potential channel formation. Despite extensive research on mammalian and plant annexins, there is limited information on annexins in invertebrates, fungi, and protists. This review seeks to bridge this knowledge gap, providing a comprehensive understanding of annexin–membrane interactions and their potential implications for cellular function and disease mechanisms across eukaryotic lineages.

## 1. Introduction

Plasma membranes, the defining feature of cellular life, serve as the critical interface between the cell and its environment. These complex structures, composed primarily of lipids, proteins, and carbohydrates, not only provide a physical barrier that delineates the cell but also play a pivotal role in numerous cellular processes. They regulate the transport of substances in and out of the cell, facilitate cell-to-cell communication, and provide a platform for biochemical reactions [1]. Within this intricate membrane landscape, certain protein families have adapted to interact specifically with the lipid components of the membrane, mediating crucial functions of the cell. One such protein family is annexins [2].

Annexins constitute a protein family found in almost every eukaryote investigated to date, including animals, plants, and fungi. Annexins are known for two main properties: the ability to bind negatively charged phospholipids and to complex with calcium ions. In the annexin structure, we can specify the disordered head region and the conserved core region containing a 70 amino acid repeat sequence known as the annexin repeat or fold [2,3,4]. Interestingly, annexin homologs have recently also been discovered in bacteria [5], expanding our understanding of the distribution of these proteins beyond eukaryotes. This discovery suggests that annexins may play a more universal role in cellular processes than previously thought.

The basic functions of annexins revolve around their interaction with membranes and calcium ions [3,6]. They are involved in various processes such as forming structures on lipid membranes that can work as anchoring points [7], which is relevant to changes in cell morphology. Annexins were also shown to be involved in the trafficking and organization of vesicles [8,9], and potentially in calcium ion channel formation [10,11,12,13] and unconventional protein secretion (UPS) [14,15].

The impact of annexins on phenotype and disease states, particularly cancer, is a growing area of research. High annexin A1 expression levels correlate with poor prognosis for survival in various cancers with limited treatment options, including triple-negative breast, pancreatic, colorectal, and prostate cancers [16]. This highlights the importance of annexins in our understanding of cellular processes and their potential as therapeutic targets [17,18,19].

The existing literature on annexin orthologues across the eukaryotic tree of life is vast. For this reason, in this review, we refer primarily to the current state of research on the annexin protein family spanning several eukaryotic taxa. The focus of this study is on membrane interactions—modes of interaction, possible structures formed on or at the membrane, and further functional implications resulting from those interactions.

## 2. Annexins: The Basics

Annexins were initially referred to as synexins [20], a term derived from the ability of annexin A7 to facilitate the fusion of lipid membranes [21]. As time progressed, various annexins were identified and named differently. For example, annexin A1 was known as lipocortin [22,23], and annexin A2 was referred to as calpactin [24,25]. Eventually, the term ‘annexin’ was introduced to denote the superfamily, and this nomenclature is now established. However, inconsistencies in naming conventions persist in the literature, particularly for abbreviations. To maintain consistency in this review, we will adhere to the official nomenclature previously proposed [4]. The abbreviation ‘Anx’ will be used for ‘annexin’, followed by a letter indicating the origin: ‘A’ for vertebrates, ‘B’ for invertebrates, ‘C’ for fungi, ‘D’ for plants, and ‘E’ for protists.

**Figure 1:**
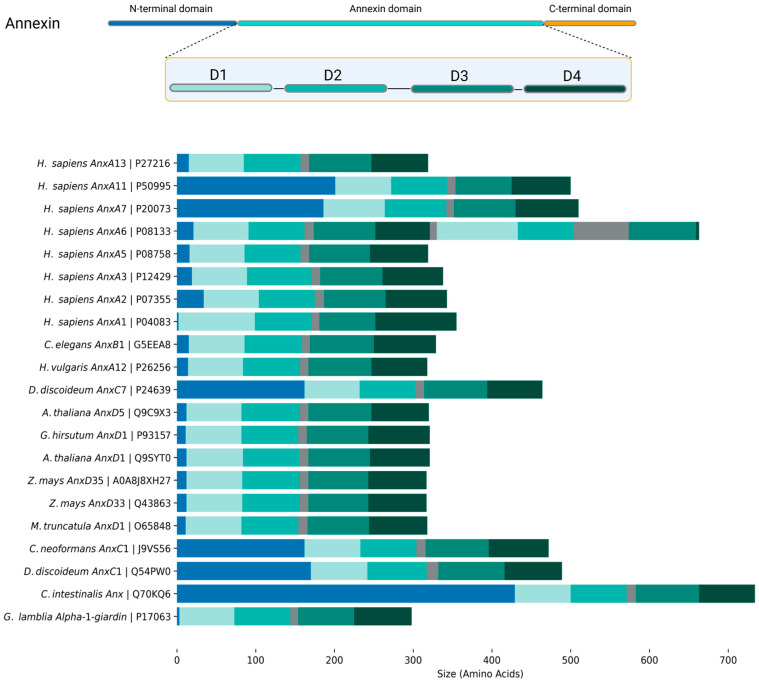
A graphical representation of a generic annexin structure with four signature domains (D1–D4), focusing on the diversity of N-terminal head domains. The figure highlights the annexins discussed in this review. Sequence information and predicted domain organization were extracted from UniProt.

Annexin structure can be coarsely separated into the N-terminal head and the C-terminal core region [2,4,26] (Figure 1). The N-terminal region, in most cases, is disordered (mostly based on in silico structural predictions). It can determine annexins’ ability to interact with other proteins or lipids, which translates into their regulatory functions. The core region is structurally conserved across all annexins and is built of four to ten repeats (Figure 1; annexin domain), consisting of five α-helices, four of which are parallel to each other and one perpendicular to them. These repeats form a curved rhombic shape with concave and convex sides. The convex side is identified by two characteristic properties of annexins: interaction with Ca^2+^ and lipids [2,27]. In comparison, the concave side takes part in interaction with the N-terminal and protein complex formation [2,4].

Annexin calcium-dependent membrane-binding properties are characterized by two types of interactions. Primary binding is defined as a calcium-mediated electrostatic interaction with phospholipid polar heads [27]. Secondary binding is based on hydrophobic interactions [27]. The specific mechanism of membrane binding varies among different annexins and is influenced by factors such as membrane lipid composition, the presence of Ca^2+^ ions, and pH [2,3,28,29]. Annexins can form various structures on the surface of a membrane, ranging from simple aggregates to complex networks, depending on the specific annexin and the conditions of the membrane [7,30,31]. These structures are speculated to contribute to the ability of annexins to regulate membrane dynamics and to participate in various cellular processes.

Following membrane binding, annexins can insert themselves into the membrane. This process is thought to involve a conformational change in annexin structure, allowing for the penetration of the lipid bilayer [2,13,32,33]. The exact mechanism of membrane insertion is still a subject of ongoing research, but is considered to play a crucial role in annexin function and subcellular location.

## 3. Annexin–Membrane Interactions: Modes and Functions

### 3.1. Annexins and Membrane Repair

#### 3.1.1. Mammalian Cell Systems

One of the most important membrane-related functions of annexins is membrane integrity and continuity. Among the annexins described in mammalian cells, AnxA1 and AnxA5 are primarily responsible for membrane repair [34,35]. AnxA1, in particular, has been identified as crucial in membrane wound healing. This cytosolic protein, when activated by micromolar Ca^2+^, binds to membrane phospholipids, thereby promoting membrane aggregation and fusion. It has been demonstrated that the resealing process can be inhibited by an AnxA1 function-blocking antibody, a small peptide competitor, and a dominant-negative AnxA1 mutant protein incapable of Ca^2+^ binding [35]. Furthermore, it has been observed that AnxA1 becomes concentrated at disruption sites marked by a resealing event. This evidence underscores the significant role for annexin, particularly AnxA1, in membrane repair and wound healing [35].

In addition to the role of AnxA1, AnxA5 has also been shown to promote membrane repair. AnxA5 is a protein that self-assembles into two-dimensional arrays on membranes upon Ca^2+^ activation. Compared to wild-type mouse perivascular cells, AnxA5-null cells exhibit a severe membrane repair defect. This defect can be rescued by the addition of AnxA5, which binds exclusively to disrupted membrane areas. Interestingly, an AnxA5 mutant that lacks the ability to form 2D arrays is unable to promote membrane repair. This suggests that the formation of 2D arrays by AnxA5 is a critical step in the membrane repair process. It is proposed that, triggered by the local influx of Ca^2+^, AnxA5 proteins bind to torn membrane edges and form a 2D array, thus promoting membrane resealing. This discovery introduces a previously unrecognized step in the membrane repair process, further highlighting the significant roles of annexins, particularly AnxA1 and AnxA5, in membrane repair and wound healing [34].

#### 3.1.2. Plant Cell Systems

In the context of plants, a study on Arabidopsis AnxD5 has shed light on its role in maintaining pollen cell membrane integrity. AnxD5, the most abundant member of the annexin family in pollen, was observed to have a transient association with the pollen membrane during in vitro hydration. When the pollinated stigma was sprayed with deionized water to simulate rainfall, AnxD5 accumulated at the pollen membrane. This suggests that AnxD5 is involved in restoring pollen membrane integrity under stress conditions. Furthermore, treatments with calcium or magnesium, which caused rupture or shrinkage of the pollen, also induced AnxD5 recruitment to the pollen membrane [36]. These findings support the model that plant annexins, like their metazoan counterparts, are involved in the maintenance of membrane integrity.

#### 3.1.3. Other Cell Systems

There is limited information available on annexins from different kingdoms. In the context of the fungus *Cryptococcus neoformans*, no alterations resulting from the deletion of AnxC1 could be observed [37]. Conversely, in Dictyostelium, the response to membrane damage was found to be slower following the deletion of annexins AnxC1 and AnxC2 [38].

### 3.2. Annexins as Calcium Ion Channels

#### 3.2.1. Mammalian Cell Systems

One of the initial proposed roles of annexins was mediating calcium ion transport through the lipid membrane, an observation based on conductance studies on recombinant human AnxA5 containing a mutation of Glu95 to Ser (E95S). These studies were performed using artificial liposomes containing phosphatidylserine (PS). The results of these studies were further substantiated by additional electrophysiological and structural investigations. The mutation E95S resulted in a lower single-channel conductance for calcium and a strongly increased conductance for sodium and potassium, indicating that Glu95 is a crucial constituent of the ion selectivity filter. Despite only minor differences in the crystal structures of mutant and wild-type AnxA5 around the mutation site, the mutant showed structural differences elsewhere, including the presence of a calcium-binding site in domain III unrelated to the mutation. Analysis of the membrane-bound form of AnxA5 by electron microscopy revealed no significant differences between the wild-type and mutant, suggesting that the observed changes in ion conductance were primarily due to the specific mutation rather than overall structural alterations [12].

#### 3.2.2. Plant Cell Systems

Research around calcium ion transport has also been conducted on plant annexins. A few examples of these annexins include ZmAnxD33/35 (annexins 33 and 35 from *Zea mays*) [39], CaAnxD24 (annexin 24 from *Capsicum annuum*) [40], MtAnxD1 (annexin 1 from *Medicago truncatula*) [41], and AtAnxD1 (annexin 1 from Arabidopsis) [42]. These annexins can form calcium-permeable transport channels and regulate cytosolic calcium levels, and ZmAnxD33/35 can allow both extracellular calcium and potassium through when applied to the cytosolic side of a planar lipid bilayer. Interestingly, the conductance created by ZmAnxD33/35 was blocked by the cation channel blocker Gd^3+^, indicating the creation of a trans-bilayer conductance. This conductance was found to be voltage-regulated and influenced by the presence of malondialdehyde, a substance produced in membranes during stress responses [39]. Moreover, MtAnxD1 showed single-channel behavior, with varying single-channel conductance based on the amount of annexin present [40]. This behavior mirrors that of animal annexins in lipid bilayers, thus highlighting the potential for these proteins in adjusting cellular responses to various stimuli and underscoring the necessity for additional research to fully comprehend their exact functions and roles in plant physiology.

#### 3.2.3. The Debate Around Annexins as True Ca^2+^ Ion Channels

**Figure 2:**
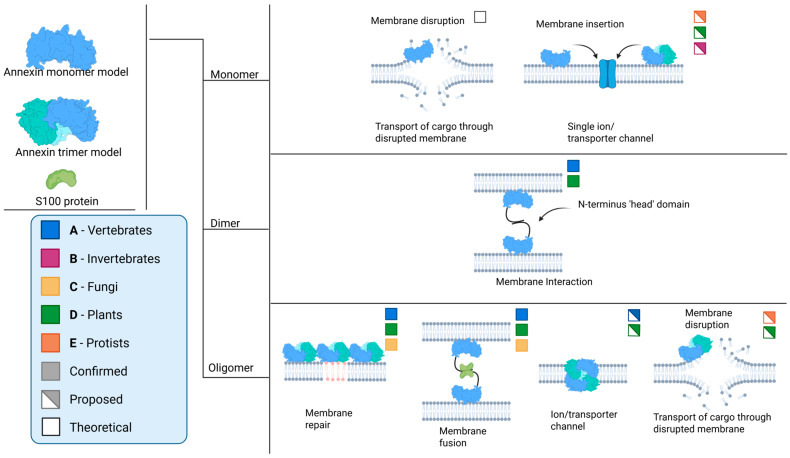
Possible and confirmed interactions, and (putative) functions, of annexins across various biological kingdoms. Information extracted and schematized from several sources [2,4,12,15,28,30,31,34,35,36,37,39,41,43,45,46,47,48,49,50,51,52,53,54,55].

Despite the data referenced above, the formation of channels by annexins remains a subject of debate [43,44]. The key question is how a peripherally associated membrane protein can facilitate the conduction of Ca^2+^ (Figure 2; monomer model). Furthermore, there are inconsistencies between the diameter of the central pore and the observed conductance values.

Two main models present the mechanism of Ca^2+^ transport by annexins. The first model, based on theoretical calculations, suggests that AnxA5 could disrupt the lipid organization in the bilayer at the site of Ca^2+^-dependent attachment to such an extent that it effectively creates a pore in the membrane, thereby allowing Ca^2+^ entry [45]. The second model, which is backed by limited experimental data, is based on extensive mutagenesis of the Hydra AnxB12. Combined with spin-labeling experiments, structural analysis of AnxB12 mutants revealed that the protein undergoes a significant conformational change at a mildly acidic pH, accompanied by membrane insertion [28]. Despite the thermodynamic implications of such a structural transformation, a hypothetically membrane-inserted annexin would possess seven transmembrane domains, thus adopting the structure of a more conventional channel [33]. However, we need to stress that there has, as yet, been no *direct* evidence for transmembrane annexin states in vivo, with a role as Ca^2+^ ion channels. This is despite extensive investigations using a range of biophysical approaches for the *direct* detection of membrane insertion. For this reason, support for annexins as true ion channels has waned over the years, with the understanding that their Ca^2+^-binding properties might cause transient Ca^2+^ transport events across the membrane, as a secondary effect [43,44].

### 3.3. Annexins and Protein Trafficking

#### 3.3.1. Mammalian Cell Systems

As previously mentioned, the name annexin originates from its function to connect/bind together proteins or membranes [2,20]. Moreover, characteristic names for each annexin were coined based on the observed interaction [2]; for example, lipocortin (AnxA1) binds steroid-inducible lipase inhibitors [22,23]; calpactin (AnxA2) complexes Ca^2+^, phospholipids, and actin [25]; and synexin (AnxA7) interacts with chromaffin granules [21].

Annexins are reported to be responsible for tethering other molecules, thereby promoting interactions and serving as regulatory units in cellular processes. For instance, AnxA1 acts as a bridge linking multivesicular bodies to the endoplasmic reticulum (ER) [46,56]. The tyrosine-phosphorylated (pTyr) form of AnxA1 provides a docking site for tyrosine phosphatase 1B, which is located in the ER, enabling the interaction of this enzyme with the activated epidermal growth factor receptor located on the membrane of multivesicular bodies [56]. Additionally, the same annexin via regulation of membrane interaction between the ER and multivesicular bodies is necessary to initiate the transport of cholesterol from the ER to endosomes [46].

Annexin-related trafficking is not limited to membrane–membrane interaction. Both AnxA11 and potentially AnxA7 are involved with lysosomes and RNA granules as they are transported to the distal regions of the axon [57]. Some annexins have been found to be responsible for the trafficking of foreign molecules in cells, such as AnxA2, which was found to organize the transport of synthetic antisense oligonucleotides from early to late endosomes [8]. AnxA1 and AnxA2 have distinct roles in the retrograde trafficking of Shiga toxin to the *trans*-Golgi network. Specifically, these proteins play a role in the uptake and intracellular transport of the bacterial Shiga toxin and the plant toxin ricin [9].

#### 3.3.2. Plant Cell Systems

The impact of annexins on trafficking and interaction with the membrane was noted in a study where researchers employed Brefeldin A, a compound known for its ability to disrupt endomembrane trafficking, which in turn inhibits pollen germination [58]. AnxD5 was revealed as a putative “linker” between Ca^2+^ signaling, the actin cytoskeleton, and the membrane. These elements are all essential for pollen development and pollen tube growth. The study further demonstrated that AnxD5 is associated with the phospholipid membrane, and it is stimulated by Ca^2+^ in vitro. Interestingly, pollen germination and pollen tube growth of plants overexpressing AnxD5 showed increased resistance to Brefeldin A treatment, and this effect was also regulated by calcium ions. This suggests that AnxD5 promotes endomembrane trafficking modulated by calcium, thereby playing a vital role in angiosperm pollen development, germination, and pollen tube growth.

#### 3.3.3. Other Cell Systems

Information about specific functions in organisms other than plants and mammals remains limited. One example is annexin from the slime mold *Physarum polycephalum* [59]. In a study by T. Shirakawa et al., it was demonstrated that *P. polycephalum* annexin can bind to two different classes of aminoacyl–tRNA synthetases [59]. The authors speculated that this interaction may impact the protein translation machinery and could also be involved in membrane fusion or other dynamic membrane processes. Impact on trafficking was also observed in *Caenorhabditis elegans* deletion mutants for AnxB1 (nex-1) [60], including the disruption of apoptotic cell engulfment [60].

### 3.4. Membrane Traversal

Annexins are a versatile family of proteins that are postulated to regulate a variety of cellular processes. These range from the regulation of protein activity [61] to the control of polysaccharide synthesis [62]. However, one property of annexins related to membranes merits mention—unconventional protein secretion (UPS) [15]. In contrast to conventional secretion, where proteins with a signal peptide are directed to the ER and then to the Golgi apparatus, and are either secreted or transported to other secretory compartments, UPS is the secretion of proteins without a signal peptide or any other feature associated with canonical secretion, with the entire process circumventing the Golgi apparatus. The absence of a specific secretory sequence significantly complicates the detection and investigation of this phenomenon.

Annexins have been reported as UPS substrates. However, the exact mechanism of how annexins are secreted remains unclear and is the subject of ongoing research, mostly on AnxA1. One hypothesis involves transporters as mediators of annexin UPS. AnxA1 was reported to be transported from the cytosol across the plasma membrane into the extracellular space by type II secretion mediated by an ATP-binding cassette (ABC) transporter [63]. These ATP-dependent membrane transporters are known to transport a variety of cargo, including ions, heavy metals, amino acids, and sugars [64]. Furthermore, the bacterial T1SS secretion system, which includes an ABC transporter as part of a larger protein complex, can translocate polypeptides, albeit only in their unfolded state [65,66], suggesting the possibility that ABC transporters may also be responsible for the secretion of leaderless proteins in mammalian cells. However, the evidence supporting this hypothesis is indirect and somewhat limited. For instance, one study found that glucocorticoid-stimulated AnxA1 externalization in pituitary folliculo-stellate cells was inhibited by glyburide, an ABC transporter inhibitor. This lent further support to a role for the ATP-binding cassette A1 (ABCA1) transporter in the secretion of AnxA1 [67]. Another study reported that AnxA1 secretion was inhibited by two ATPase inhibitors, vanadate and pervanadate [68]. However, in this study, glyburide did not inhibit AnxA1 secretion, suggesting that the mechanism for AnxA1 secretion may differ depending on the stimulant and the cell line used.

While these studies lend some support to a role for ABC transporters in AnxA1 secretion, given the average annexin size is well beyond the substrate size range of almost all ABC transporters characterized to date [69,70], there has, as yet, been no hard evidence for the direct involvement of ABCA1 or any other ABC transporter in AnxA1 secretion.

An alternative hypothesis proposes a direct interaction between annexins and a target membrane, leading to either membrane disruption or annexin insertion/traversal into/of the membrane (Figure 2; monomer). This process is akin to the mechanism described in the subsection on ion channels, which ultimately results in transport across the membrane [12,39,41]. In this scenario, the UPS of annexins could come about because of oligomerization and structural changes, allowing for the transport of these proteins across the membrane without the need for a secretory signal peptide. However, it is important to note that this is still a hypothesis, and further research is needed to fully understand the mechanisms involved in the UPS of annexins and their potential interactions with the membrane. This area of study holds significant promise for enhancing our understanding of protein secretion and cellular processes, especially in the context of annexin function in tissue and cellular homeostasis.

Evidence supporting the UPS of annexins in plants is scarce. However, there is a study that indirectly suggests the potential role of annexins in such processes [71]. Fernandez et al. compared two potato cultivars with different resistance levels to the oomycete *Phytophthora infestans*, and it was found that the resistant cultivar had a higher basal content of apoplastic hydrophobic proteins compared to the susceptible cultivar. Interestingly, annexin p34-like protein was one of the proteins that accumulated in both cultivars in response to *P. infestans* infection. This suggests that annexin-like proteins could be part of the plant’s defense mechanism against pathogens. They might be involved in the secretion of resistance proteins into the apoplast, a process that could potentially be an example of UPS. However, more direct evidence is needed to confirm the role of annexins in this process.

Another example emerges from the world of protists. Annexins from the intestinal protist parasite *Giardia lamblia* (syn. *intestinalis*, *duodenalis*) were termed alpha-giardins before their status as annexin orthologues was determined [72]. Some members of the 21-strong protein family were previously identified as putative UPS cargo, being found outside of the cell in the absence of any bona fide signal sequence for secretion [73,74,75]. A recent investigation localized these UPS-related alpha-giardins to the cytosol of the cell, as well as in close proximity to the membrane of the parasite’s peripheral endocytic compartments (PECs), membrane-bound endo-lysosomal organelles that were previously speculated to play a role in protein release [14,76,77]. Further evidence from immunoprecipitation-based approaches and the reconstruction of protein interactomes shows that these alpha-giardins build a protein complex at PECs, which includes other UPS substrates. This opens the possibility that alpha-giardins might act as both substrates *and* mediators of UPS in Giardia, in virtue of their ability to bind and possibly destabilize membranes to aid traversal [14].

## 4. Regulation of Annexin Function and Membrane Interaction

### 4.1. The Role of the N-Terminal Head Region

Annexins exhibit an overall conserved signature core structure. In contrast, the N-terminal head region exhibits significant variability and a generally disordered structure. However, it is this feature that plays an important role in determining a given annexin’s function and protein interaction partners and, in its diversity, reflects the versatile role of annexins in cells.

Annexin heads can vary from only a few residues in length, as in AnxA3, AnxD1, or alpha-1-giardin, to over 100 residues, such as in AnxA11, AnxC1, or *Ciona intestinalis* annexin (UniProtID: Q70KQ6) [4]. A documented example of this is the N-terminus of AnxA7. When it is removed, well-known AnxA7 functions such as calcium-dependent membrane binding, aggregation, and fusion are all abolished or severely diminished [47,78].

The N′-terminal head domain is the main site of interaction with other protein partners (Figure 2; dimer and oligomer models). The S100 proteins, which are dimeric EF-hand calcium-binding proteins, are known to modulate the functions of annexins through their interactions [79]. S100A10, a member of the S100 protein family, forms a tight complex with AnxA2, putatively enhancing its affinity for phospholipid membranes and promoting membrane fusion events [48]. This interaction is crucial for various cellular processes, including exocytosis and endocytosis. Other annexins, such as AnxA6, have also been found to interact with S100 proteins, suggesting a broader role for these interactions in cellular processes [49,51]. Protein–protein interactions encompass the oligomerization of annexins (Figure 2). Specifically, AnxA13, an annexin unique to the human intestine, employs an alternative folding mechanism in its head domain to achieve dimerization. This process significantly influences its ability to bind to membranes [54].

The N-terminal domain of annexins is not only involved in protein–protein interactions but also in post-translational modifications. For example, the phosphorylation of the N-terminal domain of AnxA2 regulates interaction with S100A4 and S100A10 [53]. Given the extensive diversity and ubiquitous presence of the annexin protein family and its head domains across various organisms, we still have a considerable journey ahead in uncovering all aspects of annexin regulation involving the N-terminus.

### 4.2. Calcium-Dependent Regulation and Notable Exceptions

Generally, calcium ions play a pivotal role in the regulation of annexins. The calcium-binding sites in annexins are located on the convex side of the protein, in the so-called type II calcium-binding motifs [11,80,81]. The binding of calcium ions to specific sites on the annexin molecule changes the side’s overall charge and triggers a conformational change, enabling the annexin to bind to phospholipids present in the cell membrane [11]. This calcium-induced binding is reversible and highly sensitive to changes in calcium concentration [82]. Typically, an annexin monomer contains three to four calcium-binding motifs (consensus KGXGT38(D/E)) [80,81], allowing for the fine-tuning of annexin functions in response to cellular calcium signals [80,81,82,83]. When annexin oligomers are formed, an additional level of response regulation to fluctuations in intracellular calcium levels can be achieved.

While calcium binding is a well-known characteristic of annexins, there are notable exceptions linked to pH. At lower pH, annexins interact with lipids, change their conformation, and insert themselves into the lipid membrane. This has been demonstrated in vitro with AnxA5 and A13b, which undergo a conformational change at mildly acidic pH resembling that observed after calcium binding. This conformational change seems to be an intermediate state for the calcium-independent binding of AnxA5 to phosphatidylserine (PS) liposomes at pH 4 and induces leakage from PS vesicles [84,85]. Interaction with negatively charged phospholipid vesicles at slightly low pH has also been described for AnxA4 [86], which induces leakage from these vesicles, and for AnxA6, suggesting that this insertion is a prerequisite for the formation of calcium channels [13]. A pH-driven calcium-independent insertion into PS-rich monolayers has also been observed in AnxA1 and has been suggested for AnxA5 [10,53]. In addition, studies carried out with AnxB12 from Hydra have revealed that annexins can be inserted into the lipid bilayer at acidic pH [29]. Electron paramagnetic resonance analysis showed that this region refolded and formed a continuous amphipathic α-helix after calcium-independent binding to membranes at mildly acidic pH [28]. These observations suggest the presence of a proton-dependent switch in annexins that harbors the information to induce membrane insertion.

The most notable exceptions are those of annexins that can bind to lipids independently of either Ca^2+^ or pH. Two plant annexins—AnxD24 (AnxCa32) from *Capsicum annuum* and AnxD1 from *Gossypium hirsutum*—have been studied in detail for their ability to bind liposomes in a calcium-independent fashion and at neutral pH, albeit at lower efficiency [87]. This binding is regulated by three conserved surface-exposed residues on the convex side of the proteins, which play a crucial role in membrane binding.

### 4.3. Oligomerization

Annexins are often organized in oligomers, and their properties can fluctuate based on their oligomeric state. Both oligomerization involving the N-terminal head and oligomerization involving the core could occur, based on limited experimental evidence (Figure 2; dimer and oligomer models).

In the scenario involving the head domain, there are two possible outcomes. The first is the formation of homodimers through interaction with N-terminal domains, as demonstrated in AnxA4 and AnxA5 [88]. The second outcome involves interaction with N-terminal domains and additional interaction with S100 proteins, resulting in tetramers or octamers, as seen in AnxA1 and AnxA2 [31,50,52]. In the case of AnxA2, this protein forms oligomers upon binding to membranes containing negatively charged phospholipids. This seems to be facilitated by lateral protein–protein interactions, as evidenced by the compromised oligomer formation observed in AnxA2 mutants with amino acid substitutions in residues predicted to be involved in these interactions. Despite these mutations, AnxA2 still retains its ability to bind to negatively charged membranes in the presence of Ca^2+^ [89,90]. This suggests that while lateral protein–protein interactions play a significant role in the formation of AnxA2 clusters at the membrane, they are not the sole determinant of membrane binding.

The second type of interaction between proteins is interaction within the core domain, leading to the formation of trimers. Primarily, it was described in the crystal structures of AnxA5 [30]. Using electron microscopy, AnxA5 was shown to organize in trimers at the membrane [55]. Oligomerization in annexins depends on post-translational modifications, the presence of negatively charged lipids, and calcium concentration [36,55]. These diverse interaction scenarios highlight the complex nature of annexin oligomerization and how this might be yet another level of regulation of annexin function in the cell.

## 5. Open Questions and Future Research Directions

Annexins, a diverse family of proteins, are present across a vast range of organisms and serve versatile functions. This short review demonstrates their remarkable versatility in action, properties, and interactions and underlines their crucial role in several essential cellular processes.

However, annexin diversity is truly underappreciated and under characterized. The majority of functional characterizations concern mammalian and plant annexins, with very limited investigation of B, C, and E annexin types beyond cursory analysis of their membrane-binding preferences. The mechanisms of annexin regulation are only partially understood, even in the field of mammalian annexins, with virtually no understanding of annexin regulatory mechanisms in other systems. Given that annexins are likely one of the most ancient eukaryotic protein families, with clear bacterial counterparts, it is possible to speculate that their core ability to bind membranes has evolved for adaptive deployment across different and often very diverged eukaryotic taxa. Protist parasitic species such as *G. lamblia*, *Entamoeba histolytica*, and *Trypanosoma* present significantly reduced and/or unique subcellular endomembrane compartments. Therefore, these species, and their free-living relatives, might be key to understanding conserved and perhaps ancestral features of annexin function and regulation, which might be overshadowed in more complex cellular settings. For example, Giardia annexin orthologues, the alpha-giardins, almost all present extremely short N-termini, raising questions on how such short stretches could possibly mediate both homo- and hetero-interaction, complex formation, post-translational modifications, etc., and, perhaps even more intriguingly, how this might be adaptive to a parasitic lifestyle.

In conclusion, the annexin protein family constitutes an excellent candidate for a comprehensive investigation of diversity, functionality, regulation, and protein–membrane interaction, given its almost ubiquitous distribution across eukaryotes and its involvement in essential cellular processes. Thanks to the wealth of genomic sequences derived from both targeted and shotgun genome-sequencing approaches, it is now possible to explore annexin sequence diversity in unprecedented ways. Furthermore, with a wider range of genetically tractable experimental models to explore functional conservation and divergence, the evolutionary cell biology of this ancient and important protein family might finally come of age.

## References

[1] Gatenby R.A. (2019). The Role of Cell Membrane Information Reception, Processing, and Communication in the Structure and Function of Multicellular Tissue. Int. J. Mol. Sci..

[2] Gerke V., Moss S.E. (2002). Annexins: From Structure to Function. Physiol. Rev..

[3] Rescher U., Gerke V. (2004). Annexins—Unique membrane binding proteins with diverse functions. J. Cell Sci..

[4] Moss S.E., Morgan R.O. (2004). The annexins. Genome Biol..

[5] Kodavali P.K., Dudkiewicz M., Pikuła S., Pawłowski K. (2014). Bioinformatics Analysis of Bacterial Annexins—Putative Ancestral Relatives of Eukaryotic Annexins. PLoS ONE.

[6] Liemann S., Lewit-Bentley A. (1995). Annexins: A novel family of calcium-and membrane-binding proteins in search of a function. Structure.

[7] Mo Y., Campos B., Mealy T.R., Commodore L., Head J.F., Dedman J.R., Seaton B.A. (2003). Interfacial basic cluster in annexin V couples phospholipid binding and trimer formation on membrane surfaces. J. Biol. Chem..

[8] Wang S., Sun H., Tanowitz M., Liang X., Crooke S.T. (2016). Annexin A2 facilitates endocytic trafficking of antisense oligonucleotides. Nucleic. Acids. Res..

[9] Tcatchoff L., Andersson S., Utskarpen A., Klokk T.I., Skånland S.S., Pust S., Gerke V., Sandvig K. (2012). Annexin A1 and A2: Roles in Retrograde Trafficking of Shiga Toxin. PLoS ONE.

[10] Isas J.M., Cartailler J.-P., Sokolov Y., Patel D.R., Langen R., Luecke H., Hall J.E., Haigler H.T. (2000). Annexins V and XII Insert into Bilayers at Mildly Acidic pH and Form Ion Channels. Biochemistry.

[11] Huber R., Schneider M., Mayr I., Römisch J., Paques E.-P. (1990). The calcium binding sites in human annexin V by crystal structure analysis at 2.0 A resolution Implications for membrane binding and calcium channel activity. FEBS Lett..

[12] Berendes R., Voges D., Demange P., Huber R., Burger A. (1993). Structure-Function Analysis of the Ion Channel Selectivity Filter in Human Annexin V. Science.

[13] Golczak M., Kicinska A., Bandorowicz-Pikula J., Buchet R., Szewczyk A., Pikula S. (2001). Acidic pH-induced folding of annexin VI is a prerequisite for its insertion into lipid bilayers and formation of ion channels by the protein molecules. FASEB J..

[14] Balmer E.A., Wirdnam C.D., Faso C. (2023). A core UPS molecular complement implicates unique endocytic compartments at the parasite–host interface in Giardia lamblia. Virulence.

[15] Popa S.J., Stewart S.E., Moreau K. (2018). Unconventional secretion of annexins and galectins. Semin. Cell Dev. Biol..

[16] Al-Ali H.N., Crichton S.J., Fabian C., Pepper C., Butcher D.R., Dempsey F.C., Parris C.N. (2024). A therapeutic antibody targeting annexin-A1 inhibits cancer cell growth in vitro and in vivo. Oncogene.

[17] Yan Z., Cheng X., Wang T., Hong X., Shao G., Fu C. (2022). Therapeutic potential for targeting Annexin A1 in fibrotic diseases. Genes Dis..

[18] Wang X., Shao G., Hong X., Shi Y., Zheng Y., Yu Y., Fu C. (2023). Targeting Annexin A1 as a Druggable Player to Enhance the Anti-Tumor Role of Honokiol in Colon Cancer through Autophagic Pathway. Pharmaceuticals.

[19] Li Z., Yu L., Hu B., Chen L., Jv M., Wang L., Zhou C., Wei M., Zhao L. (2021). Advances in cancer treatment: A new therapeutic target, Annexin A2. J. Cancer.

[20] Geisow M.J., Walker J.H., Boustead C., Taylor W. (1987). Annexins—New family of Ca^2+^-regulated-phospholipid binding protein. Biosci. Rep..

[21] Creutz C.E., Pazoles C.J., Pollard H.B. (1978). Identification and purification of an adrenal medullary protein (synexin) that causes calcium-dependent aggregation of isolated chromaffin granules. J. Biol. Chem..

[22] Flower R.J. (1986). Background and discovery of lipocortins. Agents Actions.

[23] Huang K.-S., Wallner B.P., Mattaliano R.J., Tizard R., Burne C., Frey A., Hession C., McGray P., Sinclair L.K., Chow E.P. (1986). Two human 35 kd inhibitors of phospholipase A2 are related to substrates of pp60v-src and of the epidermal growth factor receptor/kinase. Cell.

[24] Saris C.J.M., Tack B.F., Kristensen T., Glenney J.R., Hunter T. (1986). The cDNA sequence for the protein-tyrosine kinase substrate p36 (calpactin I heavy chain) reveals a multidomain protein with internal repeats. Cell.

[25] Glenney J.R., Tack B., Powell M.A. (1987). Calpactins: Two distinct Ca++-regulated phospholipid- and actin-binding proteins isolated from lung and placenta. J. Cell Biol..

[26] Gerke V., Gavins F.N.E., Geisow M., Grewal T., Jaiswal J.K., Nylandsted J., Rescher U. (2024). Annexins—A family of proteins with distinctive tastes for cell signaling and membrane dynamics. Nat. Commun..

[27] Bitto E., Li M., Tikhonov A.M., Schlossman M.L., Cho W. (2000). Mechanism of Annexin I-Mediated Membrane Aggregation. Biochemistry.

[28] Langen R., Isas J.M., Hubbell W.L., Haigler H.T. (1998). A transmembrane form of annexin XII detected by site-directed spin labeling. Proc. Natl. Acad. Sci. USA.

[29] Ladokhin A.S., Haigler H.T. (2005). Reversible Transition between the Surface Trimer and Membrane-Inserted Monomer of Annexin 12. Biochemistry.

[30] Oling F., Bergsma-Schutter W., Brisson A. (2001). Trimers, dimers of trimers, and trimers of trimers are common building blocks of annexin A5 two-dimensional crystals. J. Struct. Biol..

[31] Ayala-Sanmartin J., Zibouche M., Illien F., Vincent M., Gallay J. (2008). Insight into the location and dynamics of the annexin A2 N-terminal domain during Ca^2+^-induced membrane bridging. Biochim. Biophys. Acta—Biomembr..

[32] Mortimer J.C., Laohavisit A., Macpherson N., Webb A., Brownlee C., Battey N.H., Davies J.M. (2008). Annexins: Multifunctional components of growth and adaptation. J. Exp. Bot..

[33] Posokhov Y.O., Rodnin M.V., Lu L., Ladokhin A.S. (2008). Membrane Insertion Pathway of Annexin B12: Thermodynamic and Kinetic Characterization by Fluorescence Correlation Spectroscopy and Fluorescence Quenching. Biochemistry.

[34] Bouter A., Gounou C., Bérat R., Tan S., Gallois B., Granier T., d’Estaintot B.L., Pöschl E., Brachvogel B., Brisson A.R. (2011). Annexin-A5 assembled into two-dimensional arrays promotes cell membrane repair. Nat. Commun..

[35] McNeil A.K., Rescher U., Gerke V., McNeil P.L. (2006). Requirement for Annexin A1 in Plasma Membrane Repair. J. Biol. Chem..

[36] Lichocka M., Krzymowska M., Górecka M., Hennig J. (2022). Arabidopsis annexin 5 is involved in maintenance of pollen membrane integrity and permeability. J. Exp. Bot..

[37] Maryam M., Fu M.S., Alanio A., Camacho E., Goncalves D.S., Faneuff E.E., Grossman N.T., Casadevall A., Coelho C. (2019). The enigmatic role of fungal annexins: The case of Cryptococcus neoformans. Microbiology.

[38] Pervin M.S., Itoh G., Talukder M.U., Fujimoto K., Morimoto Y.V., Tanaka M., Ueda M., Yumura S. (2018). A study of wound repair in Dictyostelium cells by using novel laserporation. Sci. Rep..

[39] Laohavisit A., Mortimer J.C., Demidchik V., Coxon K.M., Stancombe M.A., Macpherson N., Brownlee C., Hofmann A., Webb A.A.R., Miedema H. (2009). Zea mays Annexins Modulate Cytosolic Free Ca^2+^ and Generate a Ca^2+^-Permeable Conductance. Plant Cell.

[40] Hofmann A., Proust J., Dorowski A., Schantz R., Huber R. (2000). Annexin 24 from Capsicum annuum. J. Biol. Chem..

[41] Kodavali P.K., Skowronek K., Koszela-Piotrowska I., Strzelecka-Kiliszek A., Pawlowski K., Pikula S. (2013). Structural and functional characterization of annexin 1 from Medicago truncatula. Plant Physiol. Biochem..

[42] Gorecka K.M., Thouverey C., Buchet R., Pikula S. (2007). Potential Role of Annexin AnnAt1 from Arabidopsis thaliana in pH-Mediated Cellular Response to Environmental Stimuli. Plant Cell Physiol..

[43] Reviakine I. (2018). When a transmembrane channel isn’t, or how biophysics and biochemistry (mis)communicate. Biochim. Biophys. Acta—Biomembr..

[44] Lizarbe M., Barrasa J., Olmo N., Gavilanes F., Turnay J. (2013). Annexin-Phospholipid Interactions. Functional Implications. Int. J. Mol. Sci..

[45] Demange P., Voges D., Benz J., Liemann S., Göttig P., Berendes R., Burger A., Huber R. (1994). Annexin V: The key to understanding ion selectivity and voltage regulation?. Trends Biochem. Sci..

[46] Eden E.R., Sanchez-Heras E., Tsapara A., Sobota A., Levine T.P., Futter C.E. (2016). Annexin A1 Tethers Membrane Contact Sites that Mediate ER to Endosome Cholesterol Transport. Dev. Cell.

[47] Naidu D.G., Raha A., Chen X.-L., Spitzer A.R., Chander A. (2005). Partial truncation of the NH2-terminus affects physical characteristics and membrane binding, aggregation, and fusion properties of annexin A7. Biochim. Biophys. Acta—Mol. Cell Biol. Lipids.

[48] Harder T., Gerke V. (1993). The subcellular distribution of early endosomes is affected by the annexin II2p11(2) complex. J. Cell Biol..

[49] Garbuglia M., Verzini M., Hofmann A., Huber R., Donato R. (2000). S100A1 and S100B interactions with annexins. Biochim. Biophys. Acta—Mol. Cell Res..

[50] Dempsey A.C., Walsh M.P., Shaw G.S. (2003). Unmasking the Annexin I Interaction from the Structure of Apo-S100A11. Structure.

[51] Chang N., Sutherland C., Hesse E., Winkfein R., Wiehler W.B., Pho M., Veillette C., Li S., Wilson D.P., Kiss E. (2007). Identification of a novel interaction between the Ca^2+^ -binding protein S100A11 and the Ca^2+^- and phospholipid-binding protein annexin A6. Am. J. Physiol.-Cell Physiol..

[52] Illien F., Finet S., Lambert O., Ayala-Sanmartin J. (2010). Different molecular arrangements of the tetrameric annexin 2 modulate the size and dynamics of membrane aggregation. Biochim. Biophys. Acta—Biomembr..

[53] Ecsédi P., Kiss B., Gógl G., Radnai L., Buday L., Koprivanacz K., Liliom K., Leveles I., Vértessy B., Jeszenői N. (2017). Regulation of the Equilibrium between Closed and Open Conformations of Annexin A2 by N-Terminal Phosphorylation and S100A4-Binding. Structure.

[54] McCulloch K.M., Yamakawa I., Shifrin D.A., McConnell R.E., Foegeding N.J., Singh P.K., Mao S., Tyska M.J., Iverson T.M. (2019). An alternative N-terminal fold of the intestine-specific annexin A13a induces dimerization and regulates membrane-binding. J. Biol. Chem..

[55] Lin Y.-C., Chipot C., Scheuring S. (2020). Annexin-V stabilizes membrane defects by inducing lipid phase transition. Nat. Commun..

[56] Eden E.R., White I.J., Tsapara A., Futter C.E. (2010). Membrane contacts between endosomes and ER provide sites for PTP1B–epidermal growth factor receptor interaction. Nat. Cell Biol..

[57] Liao Y.-C., Fernandopulle M.S., Wang G., Choi H., Hao L., Drerup C.M., Patel R., Qamar S., Nixon-Abell J., Shen Y. (2019). RNA Granules Hitchhike on Lysosomes for Long-Distance Transport, Using Annexin A11 as a Molecular Tether. Cell.

[58] Zhu J., Yuan S., Wei G., Qian D., Wu X., Jia H., Gui M., Liu W., An L., Xiang Y. (2014). Annexin5 Is Essential for Pollen Development in Arabidopsis. Mol. Plant.

[59] Shirakawa T., Nakamura A., Kohama K., Hirakata M., Ogihara S. (2005). Class-Specific Binding of Two Aminoacyl-tRNA Synthetases to Annexin, a Ca^2+^- and Phospholipid-Binding Protein. Cell Struct. Funct..

[60] Arur S., Uche U.E., Rezaul K., Fong M., Scranton V., Cowan A.E., Mohler W., Han D.K. (2003). Annexin I Is an Endogenous Ligand that Mediates Apoptotic Cell Engulfment. Dev. Cell.

[61] Hayes M.J., Shao D., Bailly M., Moss S.E. (2006). Regulation of actin dynamics by annexin 2. EMBO J..

[62] Andrawis A., Solomon M., Delmer D.P. (1993). Cotton fiber annexins: A potential role in the regulation of callose synthase. Plant J..

[63] Kuchler K., Thorner J. (1992). Secretion of Peptides and Proteins Lacking Hydrophobic Signal Sequences: The Role of Adenosine Triphosphate-Driven Membrane Translocators*. Endocr. Rev..

[64] Wilkens S. (2015). Structure and mechanism of ABC transporters. F1000Prime Rep..

[65] Morgan J.L.W., Acheson J.F., Zimmer J. (2017). Structure of a Type-1 Secretion System ABC Transporter. Structure.

[66] Akhtar A.A., Turner D.P.J. (2022). The role of bacterial ATP-binding cassette (ABC) transporters in pathogenesis and virulence: Therapeutic and vaccine potential. Microb. Pathog..

[67] Chapman L.P., Epton M.J., Buckingham J.C., Morris J.F., Christian H.C. (2003). Evidence for a Role of the Adenosine 5′-Triphosphate-Binding Cassette Transporter A1 in the Externalization of Annexin I from Pituitary Folliculo-Stellate Cells. Endocrinology.

[68] Wein S., Fauroux M., Laffitte J., de Nadaï P., Guaïni C., Pons F., Coméra C. (2004). Mediation of annexin 1 secretion by a probenecid-sensitive ABC-transporter in rat inflamed mucosa. Biochem. Pharmacol..

[69] Alam A., Locher K.P. (2023). Structure and Mechanism of Human ABC Transporters. Annu. Rev. Biophys..

[70] Thomas C., Tampé R. (2020). Structural and Mechanistic Principles of ABC Transporters. Annu. Rev. Biochem..

[71] Fernández M.B., Pagano M.R., Daleo G.R., Guevara M.G. (2012). Hydrophobic proteins secreted into the apoplast may contribute to resistance against Phytophthora infestans in potato. Plant Physiol. Biochem..

[72] Bauer B., Engelbrecht S., Bakker-Grunwald T., Scholze H. (1999). Functional identification of α-giardin as an annexin of Giardia lamblia. FEMS Microbiol. Lett..

[73] Davids B.J., Palm J.E.D., Housley M.P., Smith J.R., Andersen Y.S., Martin M.G., Hendrickson B.A., Johansen F.-E., Svärd S.G., Gillin F.D. (2006). Polymeric Immunoglobulin Receptor in Intestinal Immune Defense against the Lumen-Dwelling Protozoan Parasite Giardia. J. Immunol..

[74] Dubourg A., Xia D., Winpenny J.P., Al Naimi S., Bouzid M., Sexton D.W., Wastling J.M., Hunter P.R., Tyler K.M. (2018). Giardia secretome highlights secreted tenascins as a key component of pathogenesis. Gigascience.

[75] Ma’ayeh S.Y., Liu J., Peirasmaki D., Hörnaeus K., Bergström Lind S., Grabherr M., Bergquist J., Svärd S.G. (2017). Characterization of the Giardia intestinalis secretome during interaction with human intestinal epithelial cells: The impact on host cells. PLoS Negl. Trop. Dis..

[76] Moyano S., Musso J., Feliziani C., Zamponi N., Frontera L.S., Ropolo A.S., Lanfredi-Rangel A., Lalle M., Touz M.C. (2019). Exosome Biogenesis in the Protozoa Parasite Giardia lamblia: A Model of Reduced Interorganellar Crosstalk. Cells.

[77] Midlej V., de Souza W., Benchimol M. (2019). The peripheral vesicles gather multivesicular bodies with different behavior during the Giardia intestinalis life cycle. J. Struct. Biol..

[78] Chander A., Naidu D.G., Chen X.-L. (2006). A ten-residue domain (Y11–A20) in the NH2-terminus modulates membrane association of annexin A7. Biochim. Biophys. Acta—Mol. Cell Biol. Lipids.

[79] Rintala-Dempsey A.C., Rezvanpour A., Shaw G.S. (2008). S100–annexin complexes—Structural insights. FEBS J..

[80] Patel D.R., Isas J.M., Ladokhin A.S., Jao C.C., Kim Y.E., Kirsch T., Langen R., Haigler H.T. (2005). The conserved core domains of annexins A1, A2, A5, and B12 can be divided into two groups with different Ca^2+^-dependent membrane-binding properties. Biochemistry.

[81] Chen J.M., Sheldon A., Pincus M.R. (1993). Structure-Function Correlations of Calcium Binding and Calcium Channel Activities Based on 3-Dimensional Models of Human Annexins I, II, III, V and VII. J. Biomol. Struct. Dyn..

[82] Trotter P.J., Orchard M.A., Walker J.H. (1995). Ca^2+^ concentration during binding determines the manner in which annexin V binds to membranes. Biochem. J..

[83] Raynal P., Pollard H.B. (1994). Annexins: The problem of assessing the biological role for a gene family of multifunctional calcium- and phospholipid-binding proteins. Biochim. Biophys. Acta—Rev. Biomembr..

[84] Turnay J., Lecona E., Fernández-Lizarbe S., Guzmán-Aránguez A., Fernández M.P., Olmo N., Lizarbe M.A. (2005). Structure–function relationship in annexin A13, the founder member of the vertebrate family of annexins. Biochem. J..

[85] Sopkova J., Vincent M., Takahashi M., Lewit-Bentley A., Gallay J. (1998). Conformational Flexibility of Domain III of Annexin V Studied by Fluorescence of Tryptophan 187 and Circular Dichroism: The Effect of PH. Biochemistry.

[86] Zschörnig O., Opitz F., Müller M. (2007). Annexin A4 binding to anionic phospholipid vesicles modulated by pH and calcium. Eur. Biophys. J..

[87] Dabitz N., Hu N.-J., Yusof A.M., Tranter N., Winter A., Daley M., Zschörnig O., Brisson A., Hofmann A. (2005). Structural Determinants for Plant Annexin−Membrane Interactions. Biochemistry.

[88] Kaetzel M.A., Mo Y.D., Mealy T.R., Campos B., Bergsma-Schutter W., Brisson A., Dedman J.R., Seaton B.A. (2001). Phosphorylation Mutants Elucidate the Mechanism of Annexin IV-Mediated Membrane Aggregation. Biochemistry.

[89] Matos A.L.L., Kudruk S., Moratz J., Heflik M., Grill D., Ravoo B.J., Gerke V. (2020). Membrane Binding Promotes Annexin A2 Oligomerization. Cells.

[90] Grill D., Matos A.L.L., de Vries W.C., Kudruk S., Heflik M., Dörner W., Mootz H.D., Ravoo B.J., Galla H.J., Gerke V. (2018). Bridging of membrane surfaces by annexin A2. Sci. Rep..

